# Immunomodulation for the Treatment of Chronic Chagas Disease Cardiomyopathy: A New Approach to an Old Enemy

**DOI:** 10.3389/fcimb.2021.765879

**Published:** 2021-11-12

**Authors:** Emanuelle de Souza Santos, Dahara Keyse Carvalho Silva, Bruna Padilha Zurita Claro dos Reis, Breno Cardim Barreto, Carine Machado Azevedo Cardoso, Ricardo Ribeiro dos Santos, Cássio Santana Meira, Milena Botelho Pereira Soares

**Affiliations:** ^1^ SENAI Institute of Innovation in Health Advanced Systems (CIMATEC ISI SAS), University Center SENAI/CIMATEC, Salvador, Brazil; ^2^ Gonçalo Moniz Institute, Oswaldo Cruz Foundation (IGM-FIOCRUZ/BA), Salvador, Brazil; ^3^ School of Veterinary Medicine and Animal Science, Federal University of Bahia (UFBA), Salvador, Brazil

**Keywords:** Chagas disease, *Trypanosoma cruzi*, cardiomyopathy, immunomodulation, immunotherapy

## Abstract

Chagas disease is a parasitic infection caused by the intracellular protozoan *Trypanosoma cruzi*. Chronic Chagas cardiomyopathy (CCC) is the most severe manifestation of the disease, developed by approximately 20-40% of patients and characterized by occurrence of arrhythmias, heart failure and death. Despite having more than 100 years of discovery, Chagas disease remains without an effective treatment, especially for patients with CCC. Since the pathogenesis of CCC depends on a parasite-driven systemic inflammatory profile that leads to cardiac tissue damage, the use of immunomodulators has become a rational alternative for the treatment of CCC. In this context, different classes of drugs, cell therapies with dendritic cells or stem cells and gene therapy have shown potential to modulate systemic inflammation and myocarditis in CCC models. Based on that, the present review provides an overview of current reports regarding the use of immunomodulatory agents in treatment of CCC, bringing the challenges and future directions in this field.

## Introduction

Chagas disease, caused by *Trypanosoma cruzi* infection, is a neglected disease classically transmitted to animals and people by hematophagous triatomine vectors (Santos et al., 2020; Mansoldo et al., 2020). It represents an important public health problem, affecting around 7 million people worldwide (WHO, 2021). Although it is endemic in Latin American countries, due to international immigration, it is found nowadays in other regions, such as North America, Japan, Australia, and some countries in Europe (Losada et al., 2021).

The disease courses with an acute and a chronic phase, being, therefore, a long-lasting infection (Andrade et al., 2011). The acute phase is marked by high parasitemia and intense inflammatory response, leading to tissue damage in liver and spleen (Mills, 2020). In the chronic phase, even with the establishment of antiparasitic cellular and humoral immunities and elimination of parasites from the blood, residual parasitism persists in different tissues, including the myocardium and gastrointestinal tract (Mills, 2020). About 20–40% of patients develop digestive form and/or chronic Chagas cardiomyopathy (CCC) in a time period varying the years to decades after infection (Marin-Neto et al., 2013; Bern, 2015).

The pathogenesis of CCC involves parasite-dependent myocardial and immune-mediated tissue damage, being the most severe and frequently found form of symptomatic Chagas disease (Caldas et al., 2019). Symptoms range from mild to severe, presenting with inflammation, fibrosis, arrhythmias, and thromboembolic events, which can lead to congestive heart failure and sudden death (Rassi et al., 2017). The treatment of Chagas disease is still limited to two antiparasitic drugs, which is effective to eradicate the parasite during the acute phase of infection. Since the treatment with trypanocide agents has not yet been proven to be beneficial for patients with CCC (Morillo et al., 2015; Rassi et al., 2017), standard care to treat the symptoms of cardiac disease is provided. So far, the alternative for advanced CCC is heart transplantation, which is a limited procedure due to the availability of the organ and complications generated after immunosuppression therapy that favors the reactivation of the parasite (Morillo et al., 2015). Due to the tissue damage caused by the intense inflammatory response in CCC, an ideal therapeutic intervention should not only comprise strategies capable of eliminating the parasite, but also reducing the heart inflammation. In this context, immunomodulatory agents represent a promising approach to improve CCC treatment. Based on that, the present review provides an overview of current knowledge regarding the use of immunomodulatory agents for treatment of CCC, bringing the challenges and future directions in this field.

## Immunopathogenesis of Chagas Disease

**Figure 1:**
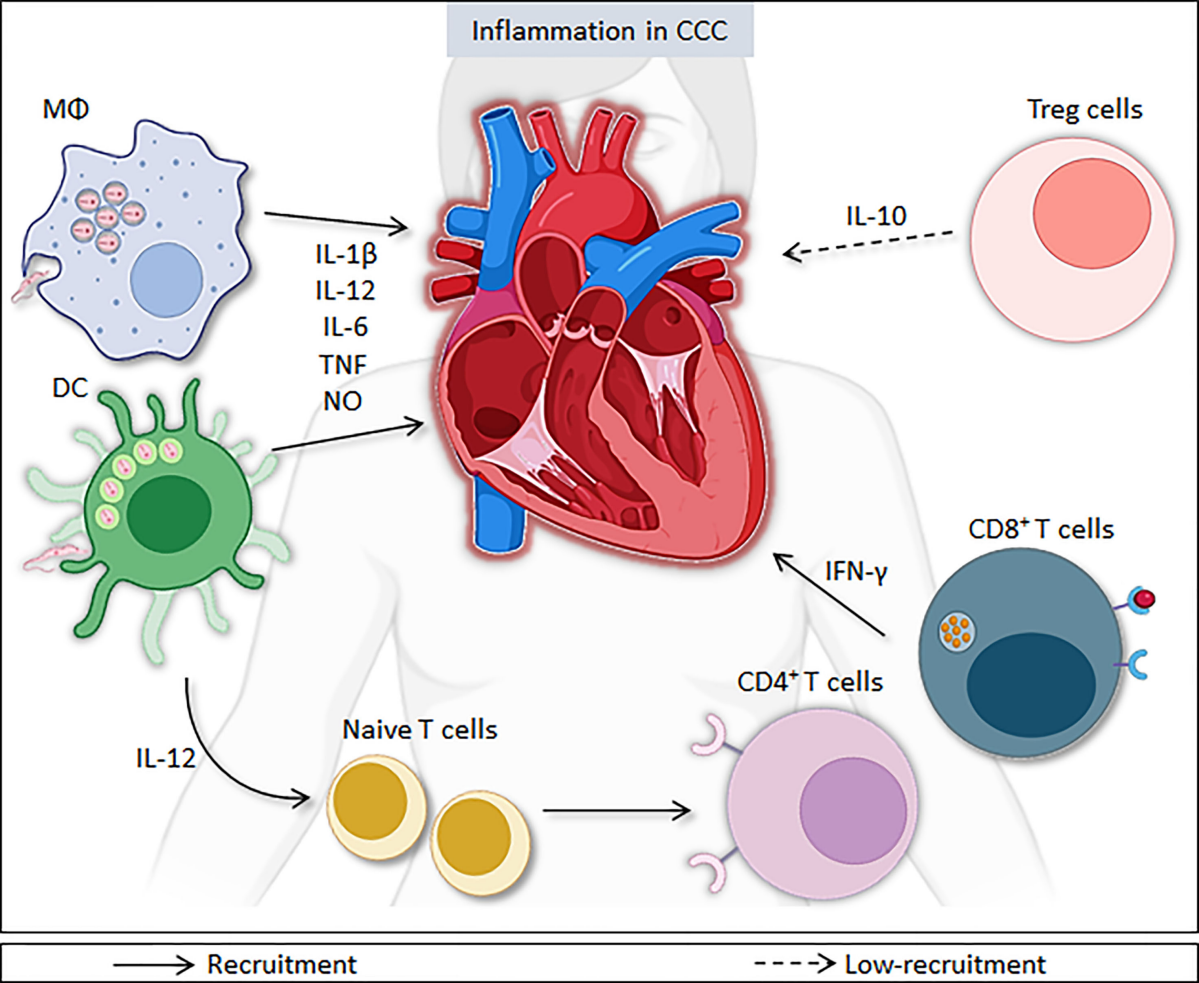
Immune responses during *Trypanosoma cruzi* infection. After activation *via* Toll-like receptors, innate immune cells, such dendritic cells and macrophages, produce pro-inflammatory molecules, such as IL-1β, IL-6, IL-8, IL-12, TNF, and nitric oxide, which help in combating the parasite. However, these molecules contribute to inflammation in the heart in both phases of disease. IL-12 production promotes a shift towards Th1 lymphocyte profile. CD4^+^ and CD8^+^ T cells, mainly through the production of IFN-γ, contribute to the activation of other immune cells and increase the production of pro-inflammatory molecules, such as IL-12, TNF, and nitric oxide, cooperating for the control of infection. The persistence of high levels of pro-inflammatory cytokines, such as IFN-γ and TNF, and the increase in IFN-γ-producing CD4^+^ and CD8^+^ T lymphocytes contribute to the persistence of inflammation in heart. Moreover, the low-recruitment of T-regulatory cells (Treg) and reduced IL-10 production also favor the persistence of heart inflammation in CCC.

During the acute Chagas disease, the first line of defense against the parasite is the innate immune system, which is crucial for *T. cruzi* elimination. Members of Toll-like receptors (TLR) and Nod-like receptors (NLR) families play important roles in the regulation of the immune response against *T. cruzi* (Pereira et al., 2014a). These receptors are involved in the recognition of molecular patterns associated with pathogens (PAMPs) and subsequent activation of innate immunity cells, which leads to modulation of the adaptive response (Pereira et al., 2014a). During the process of intracellular multiplication, the parasite releases numerous antigens, promoting the activation of the host’s immune response (Teixeira et al., 2006; Teixeira et al., 2011). Innate immune cells have TLR on their surface, which are able to recognize PAMPs, directly or indirectly inducing an immune response (Rodrigues et al., 2012; Pereira et al., 2014a). After activation *via* TLR, macrophages and dendritic cells produce pro-inflammatory molecules involved in local and systemic responses against the parasite, such as the cytokines Interleukin (IL)-1β, IL-6, IL-8, IL-12, tumor necrosis factor (TNF), and chemokines, as well as microbicidal substances, such as nitric oxide (Rodrigues et al., 2012). Macrophages and dendritic cells (DC) detect and eliminate parasites and may act as antigen presenting cells (APCs) (**Figure 1** **)**.

Macrophages play a central role the control of *T. cruzi* infection, despite representing their initial site of development (Basso, 2013; Acevedo et al., 2018). Macrophages and neutrophils produce IL-12, causing natural killer cells (NK cells) to secrete interferon-gamma (IFN-γ), which, in turn, increases the production of IL-12, TNF and nitric oxide, cooperating for the control of parasitemia. In the acute phase of infection, the stimulation of inflammatory responses is essential for the control parasitemia, requiring the action of several mediators, such as IL-12, IL-18, IFN-γ, and nitric oxide (Antúnez and Cardoni, 2000).

In the chronic phase of Chagas disease, increased frequencies of circulating T lymphocytes are found, which exert a key role in the regulation of the inflammatory process *via* secretion of pro- and anti-inflammatory mediators (Dutra and Gollob, 2008; Acevedo et al., 2018). T CD4^+^ lymphocytes release cytokines that stimulate or inhibit the action of other cells, such as macrophages, dendritic cells, and lymphocyte subpopulations, including antigen-specific B lymphocytes to produce antibodies against *T. cruzi*. There is evidence, in mouse models, that a combined response between the Th1 and Th2 profiles shows better results in parasite control, with Th1 predominating in the control and elimination process of *T. cruzi* (Silva et al., 1992; Hoft and Eickhoff, 2005; Acevedo et al., 2018). Additionally, increased frequencies of T CD8^+^ cells are usually found in places where the parasite remains, suggesting an important role of this cell population in the control of residual tissue parasitic load. Therefore, the parasite’s persistence may be due to the non-recruitment of CD8^+^ cells or to the inhibition caused by CD4^+^CD25^+^ Treg cells and TGF-β production (Tzelepis et al., 2008; Basso, 2013).

The production of TNF, IFN-γ, IL-12, IL-22, and IL-6 may vary, depending on the *T. cruzi* strain, as well as the host’s genetic background (Poveda et al., 2014; Cardillo et al., 2015). The production this cytokines can cause an exacerbated and persistent inflammatory response that induce significant damage to the host’s tissue. Therefore, in order to regulate this inflammatory process, the production of anti-inflammatory cytokines, such as IL-10 and IL-4, is induced to avoid the harmful effects that too much stimulation of the immune system could cause. Furthermore, IL-4 also plays a role in the process of stimulating the production of TGF-β, responsible for controlling the activity of antigen-presenting cells (Basso, 2013; Cardillo et al., 2015; Acevedo et al., 2018).

CCC is characterized by multifocal myocarditis, fibrosis and damage to cardiac muscle fibers, as a result of the persistence of the parasite, inflammatory cells, or both (Higuchi et al., 1987; Cunha-Neto and Chevillard, 2014). IFN-γ-producing cells are found in the hearts of mice and patients with CCC (Ferreira et al., 2014). The persistence of high levels of pro-inflammatory cytokines and the increase of IFN-γ-producing CD4^+^ and CD8^+^ T lymphocytes in peripheral blood, in addition to the reduction of Treg cells, promote tissue injury (Nogueira et al., 2014). In contrast, there is a predominance of a regulatory environment in the indeterminate chronic form, with an increase in the number of regulatory cells and elevated production of IL-10, which promotes the deactivation of macrophages and inhibits the effects of T and NK cells (Gomes et al., 2003; Cunha-Neto et al., 2009). Thus, the contribution of an exacerbated Th1 response to cardiac involvement is evident.

In order to control the infection, there must be a balance between the effector mechanisms against the parasite and the production of chemical mediators that prevent this exacerbated immune response and, consequently, tissue damage (Basso, 2013; Acevedo et al., 2018). Therefore, tuning the immune response with immunomodulatory agents may help in the prevention or control of CCC.

## Conventional Antiparasitic Therapies

Nifurtimox and benznidazole are the only drugs potentially effective against *T. cruzi* available for almost 50 years (López-Muñoz et al., 2010; Lourenço et al., 2018). However, they have efficacy limited to the chronic phase of the disease and their use is associated with side effects (López-Muñoz et al., 2010).

Nifurtimox, derived from nitrofuran - a class of drugs with antibiotic and antimicrobial activity – was developed as a therapeutic option for the treatment of Chagas disease in the 1960s (Coura and Castro, 2002). During the phase of acute infection or congenital Chagas disease, treatment with nifurtimox promotes parasitological cure in 70% of patients after two months of treatment, reducing the severity and duration of the infection and, consequently, the risk of death (Marin-Neto et al., 2009; Salomon, 2012).

The administration of nifurtimox is done orally, with recommended dosage of 8-10 mg/kg/day, for up to 90 days; however, the dosage can be adapted according to the patient’s age (Pérez-Molina et al., 2015; Pérez-Molina and Molina, 2017). On the other hand, treatment with this antiparasitic drug was discontinued in Brazil from the 1980s and later and in other South American countries, such as Argentina, Chile, and Uruguay (Coura and Castro, 2002) due to the side effects caused in about 40% of patients, including headaches, anorexia, vomiting, nausea, drowsiness, and irritability of psychiatric disorders (Benziger et al., 2017). However, these adverse effects can be reversed by reducing the dose or discontinuing treatment.

On the other hand, benznidazole, derived from nitroimidazole, has been used since the 1970s (Bern et al., 2007). Its administration has been shown to be effective for the treatment of Chagas disease in the acute phase, as well as in cases of reactivation of the infection in transplanted patients (Marin-Neto et al., 2009).

Despite presenting results similar to nifurtimox, with a parasitological cure rate of around 70%, benznidazole is considered the treatment of first choice (Salomon, 2012) due to the advantage of having a lower occurrence of adverse effects, such as allergic dermatitis, insomnia, anorexia, and weight loss (Tanowitz et al., 2009). Benznidazole is also administered orally, but its dosage varies between 5-10 mg/kg, two or three times, usually for 60 days (Pérez-Molina and Molina, 2017).

Both benznidazole and nifurtimox have excellent results in treating the acute phase or congenital infection, but its efficacy in the chronic phase still shows controversial results (Marin-Neto et al., 2009; Salomon, 2012). Nifurtimox may be harmful for adult patients who already have some type of cardiac involvement due to chronic Chagas disease, since it presents toxicity against the heart and pancreas in an experimental model (Urbina, 2010).

Although the administration of benznidazole in the chronic phase has a reduced percentage of cure, its use has been associated with the prevention of complications caused by the disease (Rassi et al., 2017), in the chronic phase, it was shown a cure rate ranging between 60-93% in children, while for adults this rate is around 40% (Andrade et al., 1996).

Aiming at developing alternatives to benznidazole and nifurtimox, inhibitors of cruzipain (or cruzain), the main cysteine protease of the parasite, and of ergosterol biosynthesis, were investigated. Among the cruzain inhibitors, the K11777 peptide stands out for its potent *in vitro* activity and for its beneficial effect on infected mice in acute and chronic infection models of Chagas disease (Engel et al., 1998; Chen et al., 2010). Unfortunately, the advance of this compound in preclinical trials was stopped due to its low tolerability in dogs and primates (Drugs For Neglected Diseases, 2014). As for compounds capable of inhibiting ergosterol synthesis, a promising example is posaconazole, which acts on the parasite’s C14α-sterol demethylase enzyme. This antifungal showed promising activity in preclinical studies, however, when tested in a phase II clinical trial, it showed a low cure rate (around 20%) in chronic chagasic individuals (Buckner and Urbina, 2012; Molina et al., 2014).

Therefore, for the chronic phase of Chagas disease, there is still no proven effective therapy. The detrimental role of inflammation in CCC, however, indicates that anti-inflammatory strategies may be beneficial in controlling the tissue damage promoted by pro-inflammatory cells. Thus, an interesting therapy for CCC may involve moderate immunosuppression, allowing the antiparasitic defenses to maintain the parasitemia control, while reducing tissue damage.

## Immunomodulatory Agents and CCC

### Drugs

As previously described, chemotherapy based on antiparasitic drugs is not able to stop or reverse the damage caused by the inflammatory response in CCC, although they can decrease or eradicate parasite load (Morillo et al., 2015). In this context, immunomodulatory drugs used alone or in combination with antiparasitic drugs can be a promising approach for the treatment of CCC. Both strategies seek to reduce inflammation, prevent organ deterioration and improve the quality of life and survival of CCC patients.

**Figure 2:**
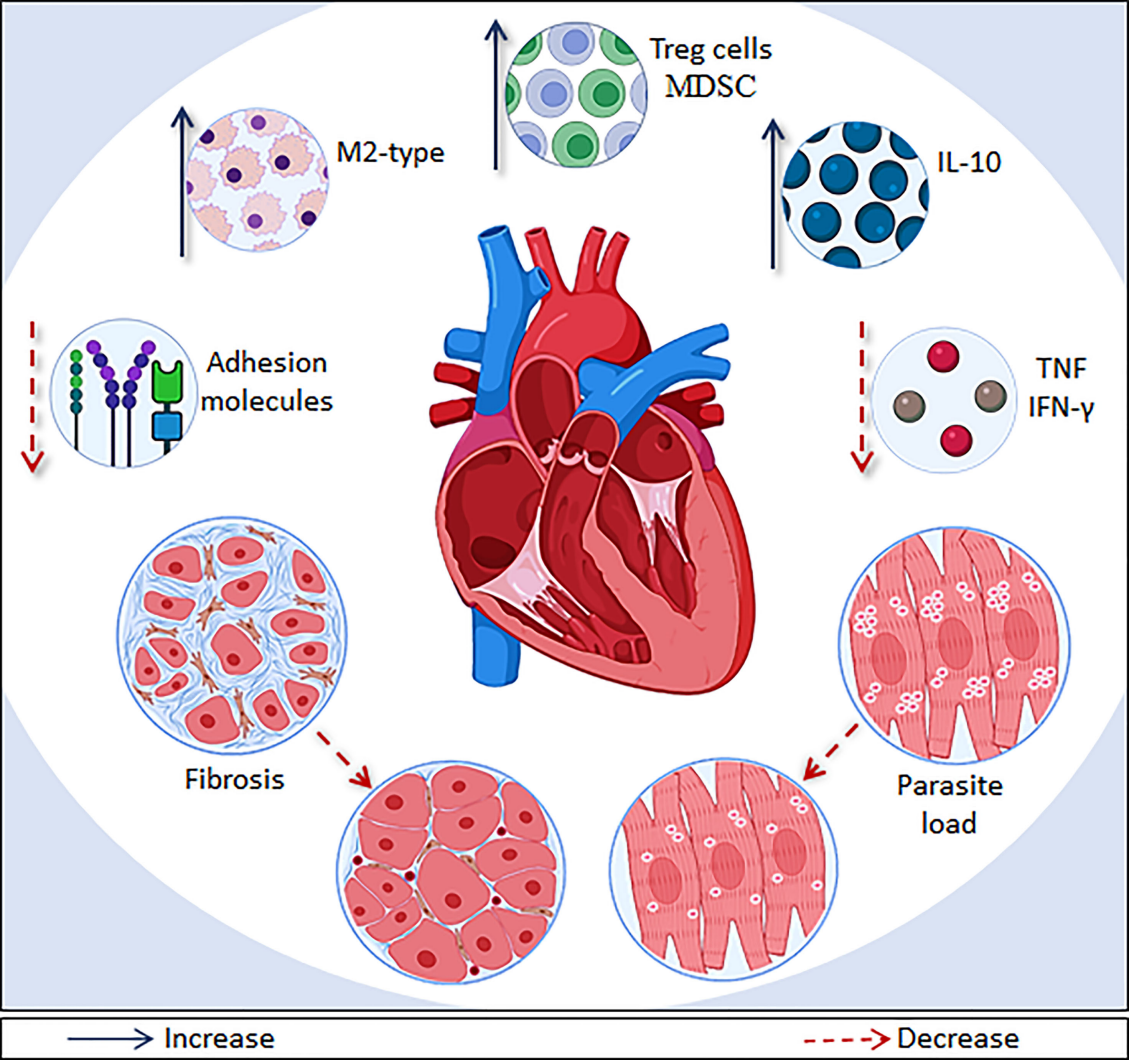
Main effects of immunomodulatory therapies on CCC models. Immunomodulatory drugs and cell/gene therapies are able to modulate systemic inflammation and myocarditis though different pathways in CCC models. The main immunomodulatory effects shown are: recruitment of T regulatory T cells (Treg) or myeloid-derived suppressor cells (MDSC); increased production of IL-10, recruitment of macrophages with a M2 phenotype; and decrease of IFN-γ, TNF, and adhesion molecules (ICAM-1, VCAM-1 and E-selectin) levels. In addition, several therapies described here also promoted a reduction of fibrosis and parasite load, which ameliorate the heart deterioration.

Several compounds of different pharmacological classes have already been shown to act as immunomodulatory agents in experimental models of mild and severe (**Table 1**). These compounds were able to modulate inflammatory process in CCC, acting in different pathways (**Figure 2**). Interestingly, none of compounds reactivated the parasitemia, proven that a moderate immunosuppression can be beneficial for CCC treatment.

**Table 1:** Immunomodulatory drugs used in CCC models.

Reference	Drug	Route/Dose	*T. cruzi* strain	Main Results
			Animal model	
Macambira et al., 2009	Granulocyte colony-stimulating factor	I.P./200 µg/Kg	Colombian	Reduction of myocarditis with increase in the number of apoptotic inflammatory cells and improvement of heart function
			C57BL/6	
Vasconcelos et al., 2013	Granulocyte colony-stimulating factor	I.P./200 µg/Kg	Colombian	Reduction of myocarditis and parasite load associated with recruitment of Treg cells
			C57BL/6	
Molina-Berríos et al., 2013	Aspirin	Oral/2 or 40 mg/Kg	Dm28c	Reduction of cardiac inflammatory infiltrates and improved of endothelial function
			BALB/c	
Pereira et al., 2015	Pentoxifylline	I.P./20 mg/Kg	Colombian	Ameliorate heart injury and dysfunction and downmodulated CD8^+^ T cells
			C57BL/6 and	
			C3H/He (H-2^k^)	
Vilar-Pereira et al., 2016	Pentoxifylline	I.P./20 mg/Kg	Colombian	Reduction myocarditis and fibrosis and improvement electrical alterations
			C57BL/6	
Cevey et al., 2017	Fenofibrate+ benzinidazole	Oral/50 to 300 mg/Kg	K-98 and RA	Reduction of myocarditis associated with reversal of the cardiac dysfunction and decrease of pro-inflammatory molecules
			BALB/c	
González-Herrera et al., 2017	Simvastatin+ benzinidazole	Oral/5 to 40 mg/Kg	Dm28c	Decrease in cardiac fibrosis and inflammation and on endothelial activation related to 15-epi-lipoxin A4
			BALB/c and Sv/129	
Vasconcelos et al., 2017	N,N-dimethylsphingosine	Oral/200 µg/Kg	Colombian	Reduction of myocarditis and parasite load associated with inflammasome pathway activation
			C57BL/6	
Meira et al., 2019	BA5 (semi-synthetic derivate from betulinic acid)	Oral/1 or 10 mg/Kg	Colombian	Decrease inflammation and fibrosis in heart associated with IL-10 production and M2 polarization
			C57BL/6	

I.P., intraperitoneal route.

Acetylsalicylic acid (aspirin), when tested in a BALB/c mouse model of chronic Chagas disease, decreased cardiac inflammatory infiltrate and cardiac fiber disarrangement (González-Herrera et al., 2017). These effects were associated with known actions of aspirin, which as reduction of thromboxane levels and adhesion molecules, such as intercellular adhesion molecule-1 (ICAM-1), vascular cell adhesion molecule-1 (VCAM-1), and E-selectin, key molecules for the recruitment of monocytes and lymphocytes, which are involved in CCC pathogenesis (Molina-Berríos et al., 2013). Interestingly, simvastatin, a cholesterol-lowering statin, also decreased myocardial inflammation alone or in combination with benznidazole, as well as the area of fibrosis in the heart. Moreover, it inhibited endothelial cells (EC) activation, as shown by the reduced expression of endothelial cell adhesion molecules, such as ICAM-1, VCAM-1, and E-selectin (González-Herrera et al., 2017). The effects of simvastatin in EC activation were partially reversed in 5-lipoxygenase knockout mice, suggesting a central role of 15-epi-lipoxin A4 production in the beneficial mechanisms of simvastatin in CCC (González-Herrera et al., 2017).

Another drug with potential use for treatment of CCC is pentoxifylline, a phosphodiesterase inhibitor with anti-inflammatory and cardioprotective effects (Sliwa et al., 2002; Shaw et al., 2009). Treatment of chagasic C57BL/6, which represents a model of mild CCC, with pentoxifylline reversed electrical abnormalities, decreased the number of inflammatory cells and reduced distances of connexin-43^+^ gap junctions in the heart. Moreover, it promoted the reduction of fibronectin area and CK-MB activity, markers of heart injury in *T. cruzi*-infected mice (Pereira et al., 2015). Interestingly, pentoxifylline also ameliorated the heart injury and electrical alterations in *T. cruzi*-infected C3H/He mice, which present a higher inflammation and heart parasitism than infected C57BL/6 mice, being considered a model of severe CCC (Pereira et al., 2014b). Furthermore, Vilar-Pereira et al. (2016) demonstrated that pentoxifylline associated with benznidazole was able to reduce the parasite load, myocarditis and fibrosis, in addition to restoring normal heart rate (QTc) corrected QT intervals in a C57BL/6 mice model for CCC.

In a different way, G-CSF, a pleiotropic cytokine, promoted a reduction of o inflammation and fibrosis in chronic chagas heart with improvement of heart function (Macambira et al., 2009; Vasconcelos et al., 2013). Correlating to the histological findings, a decrease in pro-inflammatory molecules such as, ICAM-1, galectin-3, IFN-γ, syndecan-4 and TNF was found In addition, G-CSF induced an increase of the anti-inflammatory cytokine IL-10 levels and the recruitment of Treg cells, which constitute an anti-inflammatory T-cell population (Belkaid et al., 2006). Interestingly, G-CSF also promoted a reduction in the parasite load in hearts of infected mice. Through *in vitro* experiments, a trypanocidal effect of G-CSF against *T. cruzi* was confirmed, characterizing the dual effect (antiparasitic and immunomodulatory) of this cytokine (Vasconcelos et al., 2013).

N-N-dimethylsphingosine (DMS), a pan sphingosine inhibitor, has also shown a dual nature of action in CCC, by reducing the parasitism, as well as heart inflammation and fibrosis in *T. cruzi*-infected C57BL/6 mice (Vasconcelos et al., 2017). The dual nature of DMS was supported by *in vitro* experiments showing that DMS inhibited lymphocyte proliferation, reduced nitric oxide and cytokine production in cultures of activated macrophage, having, in contrast, a direct effect on trypomastigotes and amastigotes forms of *T. cruzi*. Interestingly, DMS was shown to activate the inflammasome pathway, which contributes to its antiparasitic effect (Vasconcelos et al., 2017).

Lastly, BA5, an amide semi-synthetic derivative from the natural pentacyclic triterpenoid betulinic acid, was also proven to be a promising treatment for CCC. Initially, BA5 was characterized by its potent anti-*T. cruzi* activity, with values of IC_50_ against trypomastigotes (IC_50_ = 1.8 µM) and amastigotes (IC_50_ = 10.6 µM) lower than benznidazole (IC_50 trypomastigotes_ = 10.6 µM; IC_50 amastigotes_ = 13.5 µM) (Meira et al., 2016). In a second investigation, BA5 promoted the reduction of important inflammatory mediators, such as nitric oxide and TNF, as well as inhibition of nuclear factor-кβ (NF-кβ) in cultures of activated macrophages and also show a protective effect against a lethal dose of LPS in a mouse model of endotoxic shock and decrease edema in a delayed type of hypersensitivity model (Meira et al., 2017). Finally, in a mouse model of CCC, BA5 attenuated attenuated heart inflammation and fibrosis in the hearts of infected mice. These effects were related to a reduction of pro-inflammatory molecules, such as IFN-γ, IL-1β, and TNF, and increased IL-10 production. Moreover, polarization to anti-inflammatory/M2 macrophage phenotype was evidenced by a decrease in the expression of NOS2 and proinflammatory cytokines and the increase in M2 markers, such as Arg1 and CHI3, in mice treated with BA5 (Meira et al., 2019).

In summary, immunomodulatory compounds may be an interest therapeutic tool to the management of CCC, especially those with dual effects (antiparasitic and immunomodulatory actions) or in combination with antiparasitic drugs, such as benznidazole (Cevey et al., 2017; González-Herrera et al., 2017).

### Cell Therapy

As mentioned previously, APCs are essential elements for the immune system due to the connection established between innate and adaptive immunity and the unique ability to modulate the adaptive response, which can induce immunity or tolerance. These cells have been the target of several studies of immunotherapy aimed to promote immunomodulation (García-González et al., 2016; Moreau et al., 2017).

DCs have the ability to activate or induce T cell tolerance (Stagliano and Oppenheim, 2013), as determined by their state of maturation (Jansen et al., 2017). When mature DCs mediate immune responses in inflammatory conditions, these cells have a regulatory profile with the ability to induce immune tolerance, oppositely to DCs expressing an immature phenotype (Domogalla et al., 2017; Moreau et al., 2017).

Tolerogenic dendritic cells (tDCs) have been tested as a therapeutic tool to reduce or prevent autoimmune diseases (Hermansson et al., 2011; Lee et al., 2014; Choo et al., 2017; Huang et al., 2017; Aragão-França et al., 2018). Considering the pathogenetic mechanisms of Chagas disease and the important role of DCs in the regulation of immune responses, Santos et al. (2020) tested the therapeutic potential of tDCs in an experimental model of CCC. Administration of tDCs reduced cardiac inflammation and fibrosis, hallmarks of CCC. Furthermore, tDCs increased the frequency of Treg cells, elevated IL-10 production and inhibited the expression of markers associated with fibrosis, such as galectin-3, demonstrating a potential use of these cells in the immunotherapy for CCC.

Regenerative therapies have also been investigated for the treatment of chronic Chagas heart disease since stem cell-based therapies have emerged as an alternative to CCC treatment due to their regenerative and immunomodulatory properties (**Table 2**). Initially, bone marrow mononuclear cells (BMMC), a cell fraction containing both mesenchymal and hematopoietic stem cells, were used due to their easy obtention, low cost and long experience in their use in other diseases, facilitating translation for clinical use. In 2004, Soares et al., observed a decrease in inflammatory cell number and percentage of fibroblasts in chronically infected BALB/c and C57BL/6 mice intravenously treated with BMMC, a result that lasted up to 6 months after transplantation. Later, Goldenberg et al. (2008) demonstrated a beneficial effect of BMMC therapy in the heart function of chronically infected C-129 mice, which represents a model of right ventricular (RV) cavity dilation. The authors observed a significant reduction in the RV dilation 3 months after treatment with BMMC. Additionally, BMC treatment prevented RV dilation when applied one month after the infection.

**Table 2:** Cell therapy studies in mice models of chagasic cardiomyopathy.

Reference	Cell type/source	Route	Main results	Observation time	Mouse strain
Soares et al., 2004	Bone marrow mononuclear cells (BM-MNC)	I.V.	Reduction inflammatory cell number and fibrosis percentage	6 months	BALB/c and C57BL/6
Goldenberg et al., 2008	Bone marrow mononuclear cells (BM-MNC)	I.V.	Reduction of right ventricular dilation	3 months	C-129
Soares et al., 2011	Bone marrow mononuclear cells (BM-MNC)	I.V.	Decreased expression of genes related with inflammation and fibrosis in the heart	2 months	C57BL/6
Jasmin et al., 2012	Bone marrow mesenchymal stem cells (BM-MSC)	I.V.	Decreased right ventricular internal diameter	15-30 days	CD-1
Mello et al., 2015	Adipose derived mesenchymal stem cells (AD-MSC)	I.P.	Reduction of parasitemia, cardiac inflammation, parasitism and fibrosis Right ventricular dilation prevention	30 and 60 days	CD-1
Larocca et al., 2013	Adipose-derived mesenchymal stem cells (AD-MSC)	I.P.	Reduction of inflammation and fibrosis	2 months	C57BL/6
Silva et al., 2014	Cardiac mesenchymal stem cells (C-MSC)	I.M.	Reduction of cardiac inflammation and TNF expression	2 months	C57BL/6
Silva et al., 2018	Mesenchymal stem cells overexpressing G-CSF	I.P.	Reduction of inflammation and fibrosis; TNF and IFN-γ modulation; Increased IL-10 expression	7, 30 and 60 days	C57BL/6
Silva et al., 2018	Mesenchymal stem cells overexpressing IGF-1	I.V.	Reduction of inflammation, fibrosis and TNF expression.	2 months	C57BL/6
Santos et al., 2020	tolerogenic dendritic cells	I.P.	Reduction of inflammation, fibrosis and increased Treg cells and IL-10	3 months	C57BL/6

I.M., intramyocardial route; I.V., intravenous route; I.P., intraperitoneal route.

To investigate the mechanisms by which BMMC exerted its beneficial effects in CCC a cDNA microarray analysis was performed in hearts of chronically *T. cruzi*-infected mice, showing a large number of alterations in gene expression when compared to naïve mice (Soares et al., 2010; Soares et al., 2011). BMMC-transplanted infected mice, however, had a marked decrease in gene expression changes in the heart two months after cell therapy, showing a potent modulatory effect of BMC infusion (Soares et al., 2011). Of special interest, it was observed the down-regulation of several genes related with inflammation and fibrosis, including galectin 3, SDF-1, and TIMP-1, indicating a potent immunomodulatory action of transplanted BMMC.

The results found in the experimental models led to the development of a clinical trial to evaluate the safety and efficacy of BMC transplantation in patients with heart failure of Chagas etiology (Vilas-Boas et al., 2011). In this study, 28 patients were treated by the coronary route and evaluated 180 days later. The authors observed an improvement in the left ventricular ejection fraction, in the NYHA functional class, in the Minnesota quality of life score and in the six-minute walk test after autologous BMMC infusion, and no changes related to the procedure were found (Vilas-Boas et al., 2011). Subsequently, a randomized multicenter clinical trial was conducted with 183 patients divided into BMMC-treated and placebo groups (Santos et al., 2012). Patients were treated or not with BMMC by intracoronary route and evaluated 6 and 12 months after therapy. However, there were no statistically significant differences between groups when comparing several parameters, such as left ventricular ejection fraction (LVEF), NYHA functional class, Minnesota quality of life score, and six-minute walk test (Santos et al., 2012).

The reasons why the therapy with BMMC, despite producing good results in mice, was not observed in humans are unknown. One possible explanation may be o the differences between the mouse experimental models and the disease in humans, such as the lack of dilatation of right ventricular chamber in mice while humans present with left ventricular dysfunction. In addition, apical aneurysm and fatal arrhythmias occur in humans but not in mice, although *T. cruzi*-infected mice may show ECG changes, arrhythmias and conduction defects. Another factor to be considered is the number of cells used in the therapeutic scheme. To use in humans a dose equivalent to that used in studies with mice, it would be necessary to transplant 3.5 billion cells, while in the study performed on humans the treatment was done with 100 to 250 million cells (Carvalho et al., 2017).

In view of the above, other types of stem cells, such as mesenchymal stem/stromal cells (MSC), began to be studied as a better alternative to cell therapy in Chagas disease. MSC can be obtained from different sources, such as bone marrow, adipose tissue, and cardiac tissue, they are easy to obtain and expand, and possess immunomodulatory activity and low immunogenicity (Chamberlain et al., 2007). Such characteristics have made these cells a promising alternative for use in the treatment of CCC, due to its immunological pathogenetic mechanism.

Using bone marrow-derived MSC in the acute phase of Chagas disease, Jasmin et al. (2012) observed that, despite the low number of cells homing to the heart, MSC-treated animals had a decreased right ventricular internal diameter (RVID), suggesting a paracrine action of these cells. MSC may exert paracrine effects due to the secretion of different factors, such as growth factors, cytokines, microRNA (miRNA), and small molecules (collectively called secretome), which act in resident, as well as in immune cells, modulating their function and reducing fibrosis and inflammation, among other effects (Chang et al., 2021). When tested in the chronic phase, MSC showed similar results were observed in relation to homing and reduction of RVID (Jasmin et al., 2014). Other studies reinforced the hypothesis of paracrine effect of MSC, regardless of the source, as is the case of adipose-derived human mesenchymal stem cells (AD-MSC), transplanted by intraperitoneal route. In this case, these cells were found located in the abdominal or subcutaneous fat and, even so, the treated animals had a reduction in cardiac inflammation, parasitism and fibrosis, and prevented RV dilation (Mello et al., 2015). Additionally, Larocca et al. (2013), using the same route for MSC administration, observed a significant reduction in heart inflammation and fibrosis, but there was no improvement in arrhythmias in mice with CCC.

In another study, Silva et al. (2014) tested MSCs obtained from the mouse cardiac tissue. After treatment by intramyocardial injection in mice with CCC, a reduction in inflammation and TNF in cardiac tissue was observed two months after therapy. However, there was no significant reduction in the area of fibrosis, which correlated an increase in TGF-β expression. Although MSCs used were obtained from cardiac tissue, these cells were not able to differentiate into cardiomyocytes, since the few cells observed in cardiac tissue did not express specific cardiomyocyte markers such as troponin T and connexin 43.

Since the paracrine effects are an important mechanism of action of MSC, the genetic modification of these cells has been used as a tool to increase the production of specific factors that would enhance their therapeutic effect. Such a strategy allows bioactive molecules to be delivered systemically or directly to the injury site.

Genetic modification was carried out to induce the production of G-CSF, a growth factor known to mobilize bone marrow-derived stem cells to the peripheral blood (Anderlini and Champlin, 2008), and previously known to promote beneficial effects in the mouse model of CCC (Macambira et al., 2009; Vasconcelos et al., 2013). Silva et al. (2018) observed that G-CSF-genetically modified MSC were able to reduce inflammation and fibrosis and also reduce the production of inflammatory mediators (such as TNF and IFN-γ) and increase IL-10 levels in a more pronounced way the control MSC, when transplanted into mice with CCC. The authors attributed the observed effects to an increase in the ability to recruit suppressor cells, such as Treg cells and myeloid derived suppressor cells (MDSC).

Another growth factor tested by this approach was insulin like growth factor-1 (IGF-1), which increases the viability and differentiation of stem cells and progenitor cells (Rebouças et al., 2016). IGF-1 also improved the engraftment of MSC, which inhibits cardiomyocyte cell death in a model of myocardial infarction (Enoki et al., 2009). Using genetically modified MSC to overexpress IGF-1 (MSC-IGF-1) in a model of CCC, a reduction of inflammation and fibrosis in the heart and systemic TNF levels were found in mice treated both with IGF-1-modified or control MSC. However, MSC-IGF-1 therapy promoted a potent reduction of myofiber loss in skeletal muscle in *T. cruzi*-infected mice, compared to control groups (MSC or vehicle).

Although MSC have shown to promote beneficial effects in the mouse model of chronic Chagas heart disease, clinical trials are needed to demonstrate whether this therapy has beneficial effects in humans.

### Gene Therapy

As previously described, fibrosis and cardiac inflammation are the main features of CCC and excessive fibrosis leads to ventricular dilation and heart failure (Rassi et al., 2010; Bern, 2015; Morillo et al., 2015). Thus, seeking interventions focused on improving cardiac fibrosis could reflect an improvement in cardiac function and even a reduction in the risk of mortality (Yuan et al., 2017). Some genes related to this disease process have already been identified, but there are still gaps in relation to regulatory mechanisms (Frey and Olson, 2003).

Within the gene processes related to remodeling pathways, there is the action of microRNAs (miR), which are short 18-25 nucleotide RNA sequences that specifically regulate gene expression and apparently act as regulators of gene expression, exerting, among its functions, the inhibition of translation or promotion of mRNA (messenger RNA) degradation (Van Rooij et al., 2006; Bartel, 2009; Shukla et al., 2011).

In fact, microRNAs have been described as regulators in several pathophysiological processes, including cell proliferation, differentiation, apoptosis, and carcinogenesis. Recently, miRs have been linked to several processes regulating heart disease, such as mMiR-29, miR-30 and miR-133, described as inhibitors of collagen expression (Van Rooij et al., 2008; Duisters et al., 2009), and miR-21, shown to be an important regulator in fibroblast proliferation and fibrosis (Thum et al., 2008; Yuan et al., 2017).

In order to modulate fibrosis and inflammation present in CCC, Nonaka et al. (2021) explored the potential of miR-21 as a therapeutic target. Initially, upregulation of circulating and cardiac miR-21 was identified in patients with CCC, as well as in *T. cruzi*-infected mouse hearts. *In vitro* and well as in silico analyses showed the pro-fibrotic action of miR-21. In the same study, using a mouse experimental model of CCC, the authors showed that miR-21 blockage using nucleic acid (LNA)-anti-miR-21 caused a reduction of inflammation and fibrosis in the heart tissue. Further studies involving gene therapy are needed to explore the potential of miR-21 and other microRNAs as therapeutic candidates for CCC.

## Concluding Remarks

Despite having more than 100 years of discovery, Chagas disease remains without an effective treatment, especially for patients with CCC. Since the pathogenesis of CCC depends on a parasite-driven systemic inflammatory profile that leads to cardiac tissue damage, the use of immunomodulators has become a rational alternative for the treatment of CCC. As shown here, the use of immunomodulatory agents, such as drugs, gene and cell therapy, is able to modulate systemic inflammation and myocarditis, and promote, in certain cases, an improvement of heart function, through different pathways (**Figure 2**). In addition, the positive effects can be enhanced in therapeutic regimens that use the association of antiparasitic and immunomodulatory agents. There is a need, however, to conduct pre-clinical studies with animal models more representative to human CCC, such as non-human primates, and then clinical studies to indicate the potential clinical application of immunomodulatory agents considering the complex pathogenesis of CCC.

## Author Contributions

BB, BZ, CC, DS, CM, and ES performed the literature review and wrote the manuscript. CM, ES, RR, and MS conceived the study and wrote the manuscript. All authors contributed to the article and approved the submitted version.

## Funding

This work was supported by grants from CNPq (grant number 304258/2019-9) and FAPESB (grant number PNX0002/2014).

## Conflict of Interest

The authors declare that the research was conducted in the absence of any commercial or financial relationships that could be construed as a potential conflict of interest.

## Publisher’s Note

All claims expressed in this article are solely those of the authors and do not necessarily represent those of their affiliated organizations, or those of the publisher, the editors and the reviewers. Any product that may be evaluated in this article, or claim that may be made by its manufacturer, is not guaranteed or endorsed by the publisher.
